# Female extra-pair copulation for direct fitness benefits: A cautionary note

**DOI:** 10.1073/pnas.2207108119

**Published:** 2022-07-06

**Authors:** Jan T. Lifjeld, Tore Slagsvold

**Affiliations:** ^a^Natural History Museum, University of Oslo, 0318 Oslo, Norway;; ^b^Centre for Ecological and Evolutionary Synthesis, Department of Biological Sciences, University of Oslo, 0316 Oslo, Norway

In a field experiment with pied flycatchers, *Ficedula hypoleuca*, Krams et al. (1) showed that males assist their neighbors in antipredator defense if they have paternity in the brood. They interpreted the behavior as a direct fitness benefit to females from engaging in extra-pair copulations with a neighboring male. Here, we propose that the results can be explained without assuming female extra-pair copulation, a behavior that is rare in this species.

The experiment showed convincingly that the males’ brood defense was associated with their paternity in the neighboring brood. This is not surprising because male pied flycatchers are well-adapted to allocate parental investment between multiple simultaneous broods (2). There are basically two ways a male can obtain paternity in a neighboring nest: either through extra-pair copulation with an already-paired female or through pairing with a female that is subsequently taken over by a new male. Based on our own research on the species, we will argue that a mate-switching alternative is plausible. Males regularly defend multiple, distant nest sites (nest boxes) in order to attract a second female (3, 4). Even if the frequency of polygyny in the population is low, as indicated by Krams et al. (1), males may still perform this polyterritorial behavior (5). We have often seen males losing ownership of a second nest box to a newly arrived male, presumably because of the elevated costs of defending two or more widely separate nest boxes (3). A male will readily take over an already-paired female as long as copulations can fertilize eggs, which is from 2 d before the start of egg laying until the day the first egg is laid (6). The new male will provide parental care, and occasionally also the initial nest owner will assist in brood care (7).

There are also arguments for why an extra-pair copulation mechanism is less likely. Male pied flycatchers have small testes, the copulation rate is low, and they do not mate-guard but spend much time on polyterritorial behavior during the female’s fertile period (4, 8). All of this suggests that the male pied flycatcher shows no counteradaptation to female extra-pair copulation. Mixed-paternity broods are infrequent compared to most passerines (4, 9), and we have documented multiple cases where the female has been initially paired with the seemingly “extra-pair” sire (6, 10). If females have a direct benefit of extra-pair copulation, as suggested by Krams et al. (1), one would expect a much higher frequency of mixed-paternity broods in the absence of male paternity guards.

The only way to uncover whether a male’s paternity in the neighboring brood results from extra-pair copulation or rapid mate switching is to monitor male nest ownership during the female’s fertile period (6). We note that Krams et al. (1) did not identify the males until the nestling period when the brood defense experiment was carried out. A mate-switching origin of paternity in the neighboring brood can therefore not be ruled out, and a direct benefit of female extra-pair copulation in this species remains questionable.
